# Anticoagulant therapy for acute venous thrombo-embolism in cancer patients: A systematic review and network meta-analysis

**DOI:** 10.1371/journal.pone.0213940

**Published:** 2019-03-21

**Authors:** Anne Rossel, Helia Robert-Ebadi, Christophe Combescure, Olivier Grosgurin, Jérôme Stirnemann, Alfredo Addeo, Nicolas Garin, Thomas Agoritsas, Jean-Luc Reny, Christophe Marti

**Affiliations:** 1 Division of General Internal Medicine, University Hospitals of Geneva, 4, rue Gabrielle Perret-Gentil, CH, Geneva, Switzerland; 2 Faculty of Medicine, University of Geneva, Geneva, Switzerland; 3 Division of Angiology and Haemostasis, University Hospitals of Geneva, 4, rue Gabrielle Perret-Gentil, CH, Geneva, Switzerland; 4 Division of clinical Epidemiology, University Hospitals of Geneva, 4, rue Gabrielle Perret-Gentil, CH, Geneva, Switzerland; 5 Division of Oncology, University Hospitals of Geneva, 4, rue Gabrielle Perret-Gentil, CH, Geneva, Switzerland; Maastricht University Medical Center, NETHERLANDS

## Abstract

**Background:**

Low-molecular-weight heparin (LMWH) is usually recommended for the treatment of cancer-associated thrombosis (CAT) but this treatment requires burdensome daily injections. We did a systematic review to compare the efficacy and safety of direct oral anticoagulants (DOAC), vitamin K antagonists (VKA) and LMWH in patients with CAT.

**Methods:**

We searched Pubmed, Embase and CENTRAL for randomised controlled trials comparing DOAC, VKA and LMWH in patients with CAT. Pairwise and network meta-analyses were computed for venous thromboembolism (VTE) recurrence and bleeding complications.

**Results:**

We identified 14 studies, including 4,661 patients. In pairwise comparison, DOAC were superior to LMWH to prevent VTE recurrence (HR 0.63; 95% CI 0.42–0.96) and LMWH was superior to VKA (HR 0.53; 95% CI 0.40–0.70). The rate of major bleeding was higher with DOAC compared to LMWH (HR 1.78; 95% CI 1.11–2.87). In the network meta-analysis, DOAC had a lower, but non-significant, rate of VTE recurrence compared to LMWH (HR 0.74; 95% CI 0.54–1.01). Both DOAC (HR 0.42; 95% CI 0.29–0.61) and LMWH (HR 0.57; 95% CI 0.44–0.75) were associated with lower rates of recurrence compared to VKA. No significant difference in major bleeding rate was observed in the network meta-analysis. Inconsistency was observed between pairwise and network meta-analysis comparisons for major bleeding.

**Conclusions:**

DOAC are effective to prevent VTE recurrence in patients with CAT but are associated with an increased risk of bleeding compared to LMWH. The choice of anticoagulant should be personalised, taking into account the patient’s bleeding risk, including cancer site, and patient’s values and preferences.

## Introduction

The management of cancer-associated thrombosis (CAT) is challenging. The risk of developing a first venous thromboembolic event (VTE) in cancer patients is seven–fold higher than in the general population [1]. The risk of recurrence after a first episode of VTE is also particularly high in cancer patients after cessation of anticoagulant therapy, reaching a 12-month cumulative incidence of 20% [2]. Prolonged anticoagulant therapy is thus recommended for patients with CAT [3]. However, cancer patients are also at higher risk of bleeding due to cancer itself or to cancer-related interventions such as surgery or chemotherapy [4]. Currently, the standard of care of VTE in patients with cancer consists of subcutaneous low molecular weight heparin (LMWH), for an initial duration of 6 months, which is extended with either LMWH or VKA for an indefinite duration, as long as the cancer is not considered in remission [3]. This recommendation is based on randomised studies showing a reduced risk of VTE recurrence in cancer patients receiving LMWH compared with antivitamin K therapy [5–8]. This benefit was shown to be consistent in several meta-analyses [9–11]. However, LMWH treatment is burdensome, both from an economic and patient perspective as LMWH is expensive and requires daily injections which may further alter quality of life of cancer patients.

Due to the limited tolerance to daily injections in some patients, and to the lack of evidence to recommend LMWH beyond the initial 6 months, VKA are sometimes used in cancer patients [12]. However, the efficacy and safety of VKA may be altered by the difficulties to keep patients with cancer in the therapeutic range. Indeed, VKA therapeutic range is narrow, and VKA are subject to pharmacokinetic and pharmacodynamic interactions which are more numerous in cancer patients than in the general population. Moreover, anorexia or vomiting during chemotherapy can impair regular Vitamin K intake and absorption [13]. The low time in therapeutic range (TTR) obtained even in the setting of clinical trials is an objective reflection of this challenging issue.

DOAC have recently emerged as an alternative to VKA and LMWH for the treatment of VTE in non-cancer patients. Large-scale phase III non-inferiority trials confirmed the efficacy of these molecules compared to VKA to prevent VTE recurrence, with a similar or even more favourable safety profile in terms of bleeding events [14–18]. Previous meta-analyses [10, 11, 19, 20] based on subgroup analyses of these phase III trials concluded that DOAC are effective and safe for the treatment of CAT. However, two main limitations preclude any firm conclusions. First, only a small fraction of the studied population had cancer and they were highly selected and not representative of the general oncologic population. This is well reflected by the surprisingly low VTE recurrence rate for cancer patients, compared to the known high recurrence rate reported in observational studies. Second, these studies compared DOAC with a regimen of 5 days of LMWH followed by VKA, which is not the recommended treatment for CAT.

Recently, two randomised controlled trials (RCT) comparing DOAC to LMWH in cancer patients have been published [21, 22].

In the Hokusai cancer trial [21], edoxaban was shown non-inferior to dalteparin to prevent a composite endpoint including VTE recurrence or major bleeding. Similarly, the SELECT-D trial [22] compared Oral Factor Xa Inhibitor Rivaroxaban to dalteparin. The rate of VTE recurrence was lower in patients allocated to Rivaroxaban, but the rate of clinically relevant non major bleeding (CRNMB) was increased.

In order to further assess the evidence on the efficacy and safety of DOAC for the treatment of VTE in cancer patients, we performed a systematic review and network meta-analysis to compare VKA, LMWH and DOAC. In the presence of a limited number of studies directly comparing DOAC to the recommended standard of care (LMWH), network meta-analysis allows to provide direct and indirect comparison between DOAC and LMWH and evaluate their consistency.

## Materials and methods

### Protocol and registration

Search strategy, study selection, data extraction and analysis were performed according to a pre-defined protocol (available on request) and reported according to the PRISMA guidelines. (S1 and S2 Tables)

### Eligibility criteria

We included RCTs comparing the following anticoagulant regimens in adult patients with CAT: LMWH vs VKA, DOAC vs VKA, DOAC vs LMWH. Studies were eligible if patients with cancer were included, even as a subgroup, if results were reported separately. Index VTE events were defined by the presence of an objectively confirmed acute deep vein thrombosis (DVT) and/or pulmonary embolism (PE). DVT had to be diagnosed either by lower limb venous compression ultrasound (CUS) or venography. PE had to be diagnosed either by computed tomography pulmonary angiography, high-probability pulmonary ventilation/perfusion scan, pulmonary angiography, or the presence of a proximal DVT on CUS in a patient with clinically suspected PE.

Thrombotic and haemorrhagic outcomes had to be objectively confirmed and adjudicated, using the same criteria as above for recurrent VTE and the widely accepted ISTH criteria for major bleeding and clinically relevant non-major bleeding.

Studies comparing various regimens of the same anticoagulant, or other classes of anticoagulant therapy such as pentasacharides or unfractionated heparin (UFH), or with a follow-up duration less than 3 months were excluded. As the definition of active cancer may vary in each study, we used the definition applied in each study. When various definitions were reported in the same study, we extracted data from patients with cancer defined as active rather than patients with cancer history or incidental cancers.

### Information sources

Two authors (AR, CM) systematically searched Pubmed, Embase and the Cochrane register of controlled Trials (CENTRAL) databases using a comprehensive search strategy without language restriction. The detailed search strategy is available in the supplementary appendix (S2 Table). To ensure a comprehensive literature search, we also examined reference lists from retrieved articles and reference literature (guidelines and systematic reviews).

### Study selection and data extraction

Two investigators (AR, CM) independently evaluated studies for possible inclusion. Non-relevant studies were excluded based on title and abstract. For potentially relevant studies, full-text was obtained and two investigators (AR, CM) independently assessed study eligibility and extracted the data on study design, patient characteristics and outcomes. Disagreement about study inclusion or data extraction was resolved by consensus or by discussion with a third author (HRE).

### Outcomes

The main outcome was the rate of objectively confirmed new VTE event. VTE events had to be diagnosed as described in the eligibility criteria.

Safety outcomes were the rate of major bleeding, clinically relevant non-major bleedings (CRNMB), all-cause mortality and gastro-intestinal (GI) bleeding.

According to the International Society of Thrombosis and Haemostasis, major bleeding is defined as a decrease in haemoglobin level of ≥2g/dl, transfusion of ≥2 units of packed red blood cells, bleeding that occurs in a critical site (intracranial, intra-spinal, intraocular, pericardial, intra-articular, intramuscular with compartment syndrome, or retroperitoneal) or fatal bleeding [23]. CRNMB is defined as overt bleeding that does not meet the criteria for major bleeding but is associated with medical intervention, unscheduled contact with a physician, interruption or discontinuation of study drug, or discomfort or impairment of activities of daily living [24].

The longest follow-up duration was used. According to inclusion criteria, minimum follow-up was 3 months.

### Risk of bias

We used the quality criteria of the Cochrane Collaboration’s tool [25] to assess risk of bias in the following domains: selection bias (random sequence generation, allocation concealment), performance bias (blinding of participants and personnel), detection bias (blinding of outcome assessment), attrition bias (incomplete outcome data), reporting bias (selective reporting), and other biases.

Two investigators assessed study quality independently. Disagreements were resolved by consensus or by a third reviewer if no consensus was found.

### Data analysis

For all outcomes, pooled risks were obtained for the different treatment arms using random effect models. Hazard ratios (HR) for comparison between treatment arms were pooled across studies using network meta-analyses with random effects [26]. Estimates were obtained by using a frequentist method with the package netmeta for R program version 3.5.0 [27]. In addition, to further assess the inconsistency of the network, the direct evidence was obtained for each pair of compared treatments by combining only studies directly comparing the two treatments. For this purpose, a model with random effects (DerSimonian and Laird approach) was used.

When HRs were not reported in original publications, they were estimated by the ratio of logarithms of event-free proportions calculated from the reported sample sizes and the number of events [28]. In order to assess the reliability of this approximation method, we computed estimates also in studies providing HRs and compared the calculated results to the actual reported HRs.

To facilitate the interpretation of pooled HRs, the effect sizes were expressed as absolute one-year risk difference (RD) between treatment arms. For this purpose, we used the formula of the ratio of logarithms of event-free proportions with the risks in LMWH arm of the Hokusai-cancer VTE study as baseline risks as this study was the largest and most recent RCT dedicated to VTE treatment in patients with cancer [21].

Heterogeneity was detected using the test based on Cochran’s Q statistic with a 10% level of statistical significance (P<0.1) and I^2^>50%. In the network meta-analysis, the generalized Cochran/s Q statistic was used to test heterogeneity and inconsistency with a 10% level of statistical significance due to the lack of power of this type of tests [29]. A sensitivity analysis was performed to evaluate the strength of the pooled HRs, by the leave-one out principle.

The two-sided significance level was 0.05 for all analyses (except for the heterogeneity and inconsistency tests). In order to determinate treatment rankings for efficacy and safety outcomes, P-scores were derived from the network meta-analysis. The P-score can be interpreted as the mean extent of certainty that treatment is better than another treatment, averaged over all competing treatments and can be seen as the frequentist equivalent of surface under the cumulative ranking (SUCRA) value [30].

## Results

### Study selection and characteristics

**Fig 1:**
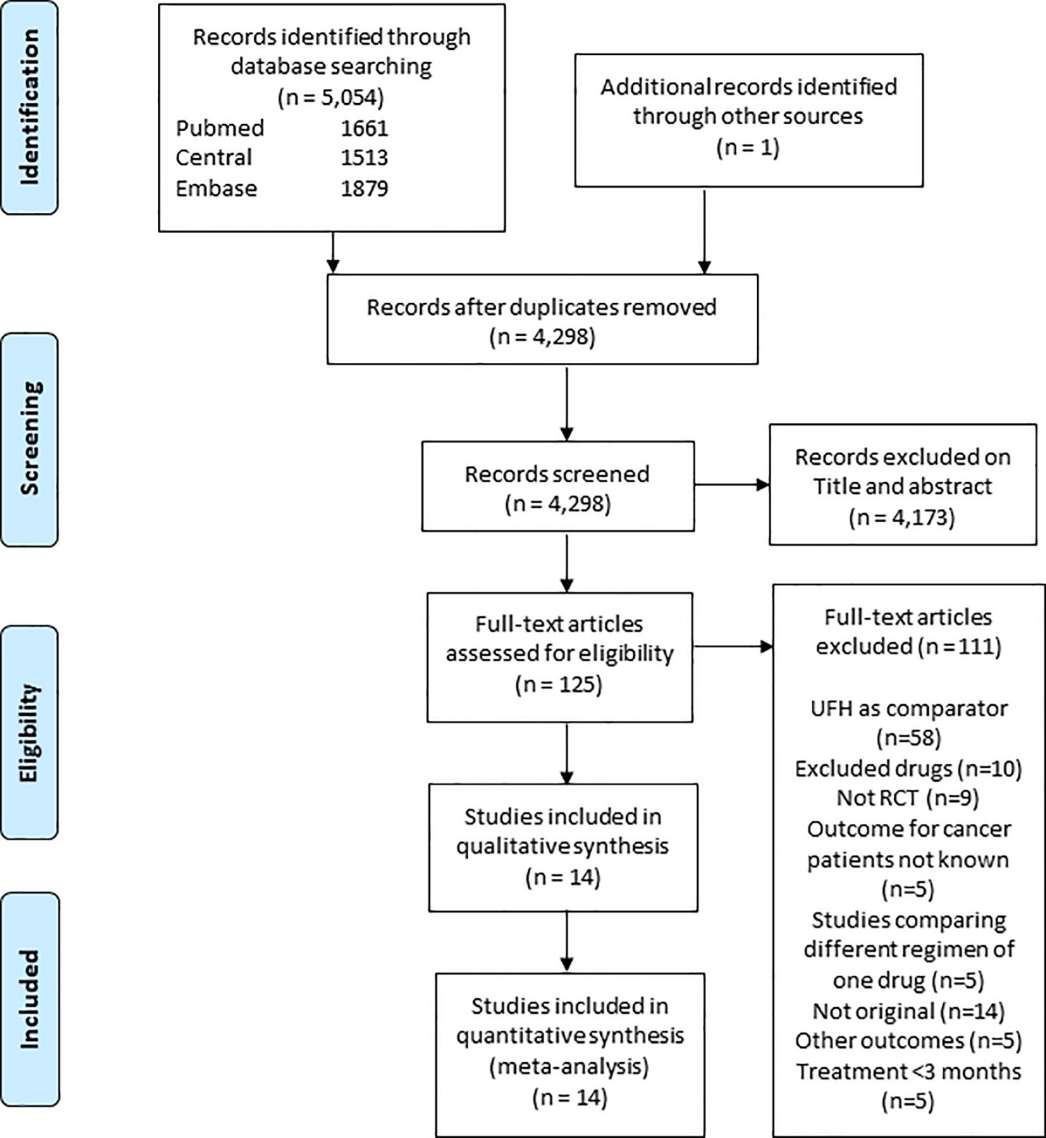
Study flow chart.

**Fig 2:**
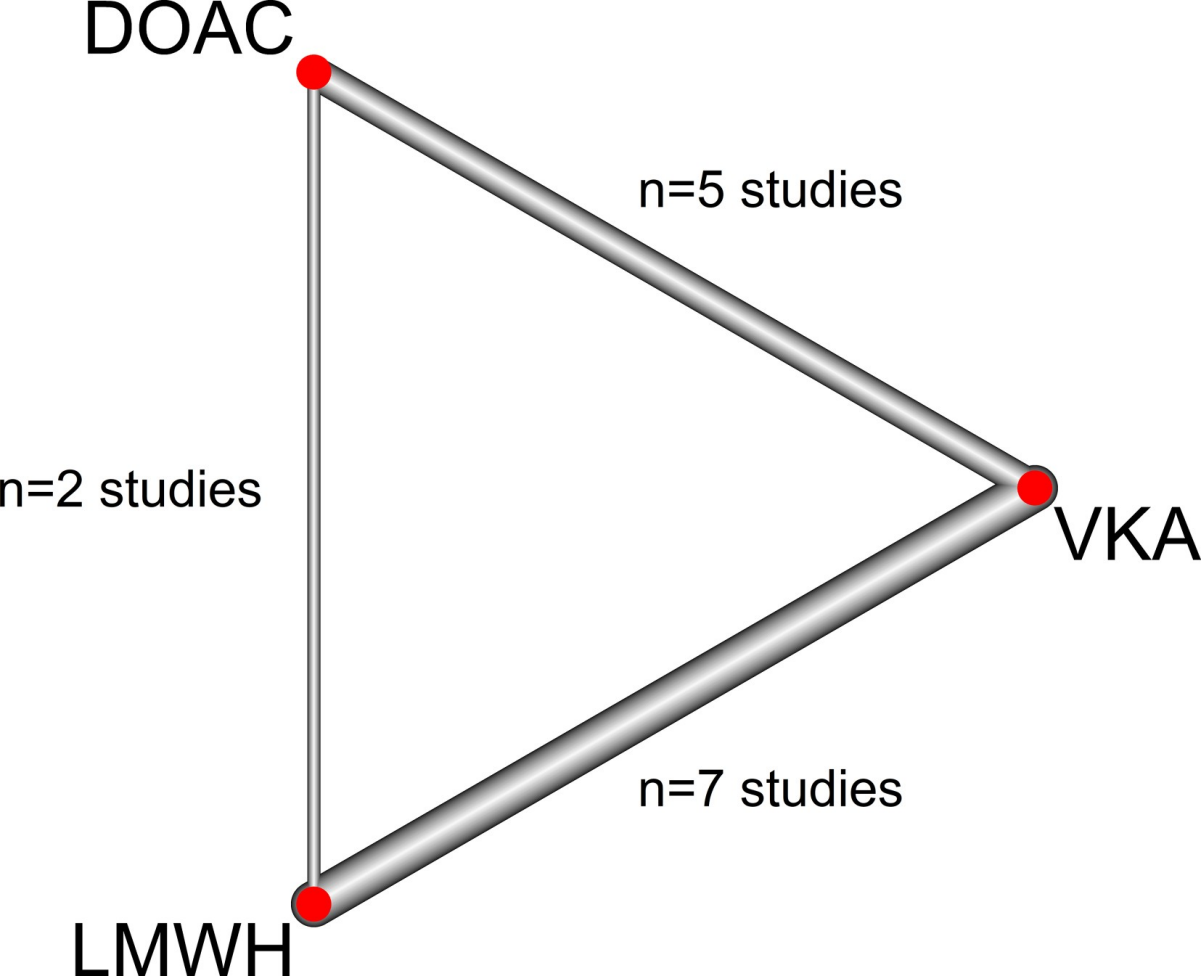
Diagram of the network.

The search retrieved 5054 references, among which 756 were duplicates (Fig 1). Fourteen RCTs including 4,661 patients were included in the meta-analysis. Seven studies [5–8, 31–33] compared LMWH to VKA (2,095 patients), 5 studies compared DOAC to VKA [34–38](1,114 patients) and the 2 remaining [21, 22] (1,452 patients) compared DOAC to LMWH. Four studies comparing DOAC to VKA, consisted of subgroup analyses of patients with cancer, included in studies for VTE treatment in a general population. Characteristics of included studies are provided in Table 1. The diagram of the network meta-analysis is provided in Fig 2.

**Table 1:** Characteristics of included studies.

Author (Study name) year	Study drug	Dose	Comparator	Dose	Treatment duration	TTR (%)	Included patients	Recurrence study drug vs comparator (%)	Major Bleeding study drug vs comparator (%)	CRNMB study drug vs Comparator (%)	Death study drug vs Comparator (%)
Raskob (Hokusai-VTE Cancer) 2018[21]	LMWH 5–7 days, then Edoxaban	60mg or 30mg/d	Dalteparin	200UI/kg /d during 30days, then 150UI/kg/d	Min. 6months	NA	1046	7.9 vs 11.3	6.9 vs 4.0	14.6 vs 11.1	39.5 vs 36.6
Young (Select-D) 2018[22]	Rivaroxaban	15mg bd 3 weeks, then 20mg/d	Dalteparin	200UI/kg/d during 30days, then 150UI/kg/d	Min. 6months	NA	406	3.9vs 8.9	5.4 vs 3.0	12.3 vs 3.4	23.6 vs 27.6
Agnelli (Amplify) 2015[34]	Apixaban	10mg bd 7d, then 5mg bd	Enoxaparin-Warfarin*	1mg/kg bd min 5d, then for INR 2–3	6months	NA	159	3.7 vs 6.4	2.3 vs 5.0	10.3 vs 17.5	6 .0vs 7.7
Mazilu 2014[38]	Dabigatran	150 mg bd	LMWH-Acenocoumarol*	Target INR 2–3	6months	NA	46	0 vs 0	NA	NA	0 vs 4
Prins (EINSTEIN-DVT/PE) 2014[35]	Rivaroxaban	15mg bd 3 weeks, then 20mg/d	Enoxaparin-Warfarin*	1mg/kg bd min 5d, then for INR 2–3	3, 6 or 12 months	57%	462	2.3 vs 3.9	1.9 vs 3.9	11.6 vs 13.2	14.7 vs 11.8
Raskob (Hokusai-VTE) 2016[36]	Edoxaban	60mg/d or 30mg/d	LMWH-Warfarin*	Target INR 2–3	Min. 3 months	63%	208	3.6 vs 7.0	4.6 vs 3.0	14.7vs 23.2	28 .4vs 26.3
Schulman (RE-COVER) 2015[37]	Dabigatran	150mg bd	LMWH-Warfarin*	Target INR 2–3	6 months	48%	221	3.5 vs 4.7	3.8vs 3.0	9.5 vs 9.0	14.0 vs 15.0
Deitcher (ONCENOX) 2006[31]	Enoxaparin	1mg/kg bd 5d then 1 or 1.5mg/ kg/d	LMWH-Warfarin*	Target INR 2–3	6 months	NA	91	6.6 vs 10	9.0 vs 2.9	58.2 vs 50.0	32.8 vs 32
Hull (LITE) 2006[6]	Tinzaparin	175U/kg/d	LMWH-Warfarin*	Target INR 2–3	3 months	NA	200	7.0 vs 16.0	7.0 vs 7.0	20.0 vs 17.0	47.0 vs 47.0
Lee (CLOT) 2003[5]	Dalteparin	200U/kg /d	LMWH-VKA*	Target INR 2–3	6 months	46%	673	8.0 vs 15.8	5.6 vs 3.6	14.0 vs 19.0	38.7 vs 40.5
Lee (CATCH) 2015[8]	Tinzaparin	175U/kg/d	LMWH-Warfarin*	Target INR 2–3	6 months	47%	900	6.9vs 10.0	2.7 vs 2.4	10.9 vs 15.3	33.4 vs 30.6
Lopèz-Beret 2001[33]	Nadroparin	1,025U/10kg bd	LMWH-Acenocoumarol*	Target INR 2–3	6 months	NA	35	2.5 vs 9.0	0 vs 5.2	4.9 vs 0	11.1 vs 7.8
Meyer (CANTHANOX) 2002[7]	Enoxaparin	1mg/kg once daily	LMWH-Warfarin*	Target INR 2–3	3 months	41%	146	3 vs 4	6.7 vs 16	NA	11.3 vs 22.7
Romera 2009[32]	Tinzaparin	175U/kg once daily	LMWH-Acenocoumarol*	Target INR 2–3	6 months	NA	69	6 vs 21	NA	NA	NA

VKA: Vitamin K antagonists, d: day, bd: bi-daily, TTR: Time in the therapeutic range

* LMWH for min 5 days overlapped and followed by VKA

NA Not applicable.

### Venous thromboembolism recurrence

Fourteen studies including 4,661 patients reported VTE recurrence (Table 1) [5–8, 21, 22, 31–38]. Recurrence occurred in 4.1% (66/1310) of patients allocated to DOAC, 8.3% (151/1,797) allocated to LMWH and in 9.5% (156/1554) of those allocated to VKA. (S3 Table)

**Fig 3:**
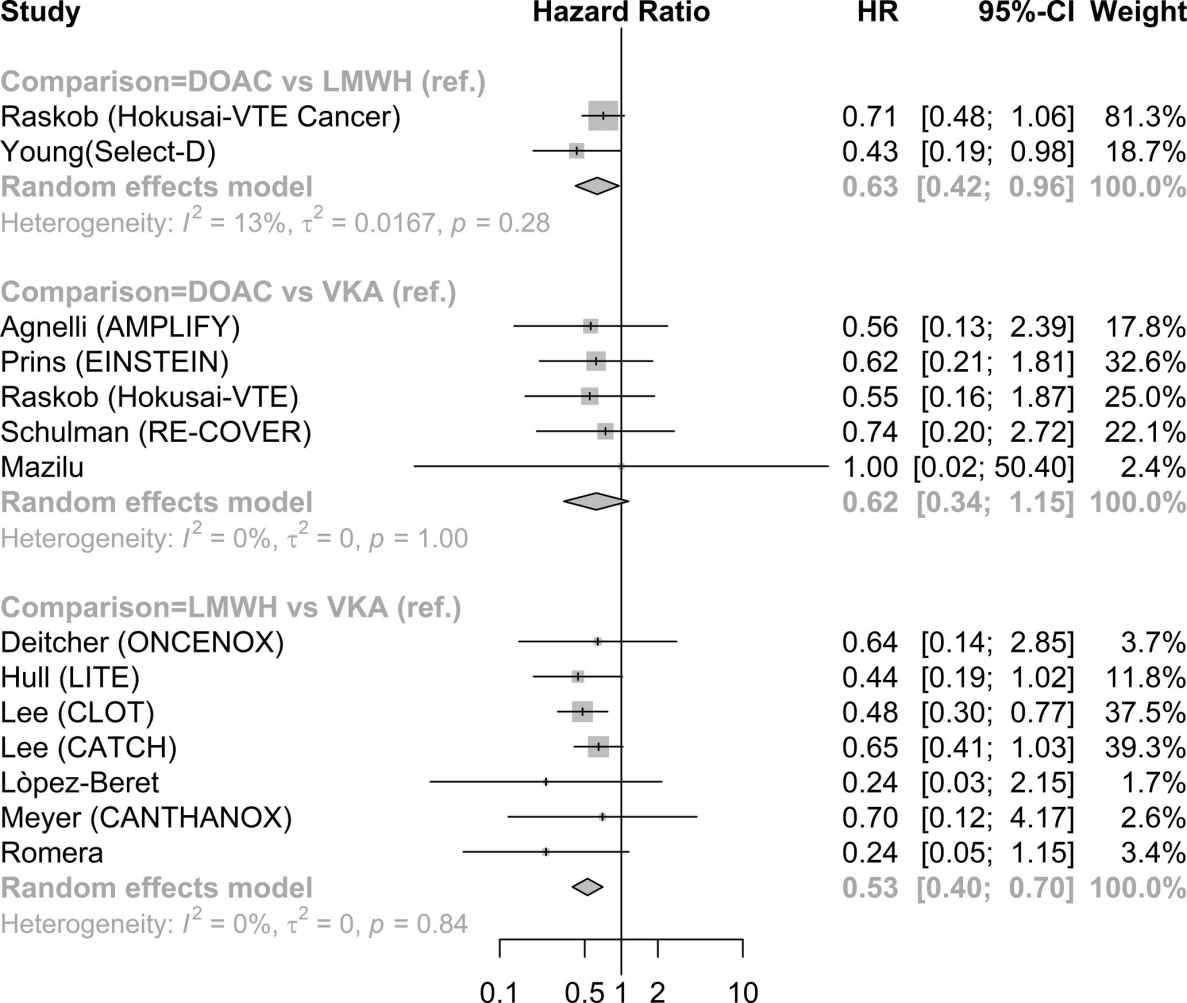
Direct meta-analysis recurrence forest plot.

In direct comparison (Table 2), treatment by DOAC was associated with a decreased rate of recurrence compared to LMWH (HR 0.63; 95%CI 0.42–0.96, RD -3.8; 95%CI -6.0 to -0.9: Table 2, Fig 3 and S4 Table). There was no significant difference between DOAC and VKA (HR 0.62; 95%CI 0.34 to 1.15). The rate of recurrence was lower in patients treated by LWMH compared to patients treated by VKA (HR 0.53; 95%CI 0.40–0.70, RD -8.9; 95% CI -14.6 to-4.4. In the network meta-analysis patients treated by DOAC had a lower, though no longer statistically significant, rate of VTE recurrence compared to LMWH (HR 0.74; 95%CI 0.54–1.01). Both DOAC (HR 0.42; 95%CI 0.29–0.61) and LMWH (HR 0.57; 95%CI 0.44–0.75) were associated with a lower rate of recurrence compared to VKA. (Table 2)

**Table 2:** Direct and network estimates.

		Direct comparisons			Network meta-analysis		
	Nb studies	Pooled HR (95%CI)	*P*	I2 (%)	Pooled HR (95%CI)	*P*	Proportion of direct evidence
***Recurrence***							
LMWH vs VKA	7	0.53 (0.40 to 0.70)	<0.001	0	0.57 (0.44 to 0.75)	<0.001	0.86
DOAC vs VKA	5	0.62 (0.34 to 1.15)	0.128	0	0.42 (0.29 to 0.61)	<0.001	0.36
DOAC vs LMWH	2	0.63 (0.42 to 0.96)	0.030	13	0.74 (0.54 to 1.01)	0.058	0.78
***Major bleeding***							
LMWH vs VKA	6	0.96 (0.57 to 1.63)	0.888	23	0.82 (0.53 to 1.27)	0.369	0.79
DOAC vs VKA	4	0.76 (0.38 to 1.53)	0.440	0	1.13 (0.66 to 1.93)	0.652	0.52
DOAC vs LMWH	2	1.78 (1.11 to 2.87)	0.017	0	1.38 (0.84 to 2.27)	0.205	0.68
***CRNMB***							
LMWH vs VKA	5	0.82 (0.52 to 1.29)	0.392	67	0.71 (0.49 to 1.04)	0.077	0.79
DOAC vs VKA	4	0.83 (0.55 to 1.27)	0.397	23	1.02 (0.67 to 1.57)	0.919	0.69
DOAC vs LMWH	2	2.11 (0.80 to 5.58)	0.131	79	1.44 (0.91 to 2.29)	0.123	0.52
***GI bleeding***							
LMWH vs VKA	2	0.42 (0.12 to 1.48)	0.177	0	0.42 (0.12 to 1.48)	0.177	1.00
DOAC vs VKA	0				1.22 (0.30 to 4.96)	0.781	0.00
DOAC vs LMWH	2	2.88 (1.53 to 5.44)	0.001	0	2.88 (1.53 to 5.44)	0.001	1.00
***Mortality***							
LMWH vs VKA	6	0.99 (0.85 to 1.16)	0.941	6	0.96 (0.83 to 1.10)	0.554	0.80
DOAC vs VKA	5	0.91 (0.68 to 1.23)	0.544	5	1.04 (0.89 to 1.22)	0.624	0.28
DOAC vs LMWH	2	1.04 (0.80 to 1.33)	0.789	2	1.08 (0.99 to 1.19)	0.089	0.92

HR: Hazard ratio, LMWH: Low molecular weight heparin, VKA: Vitamin K antagonists, DOAC: Direct oral anticoagulants, CRNMB: Clinically relevant non major bleeding, Nb: Number.

### Major bleeding

Twelve studies including 4546 patients reported major bleeding [5–8, 21, 22, 31, 33–37].

The pooled risk of major bleeding was 4.4% (63/1283) among patients receiving DOAC, 4.7% (76/1769) among patients treated with LMWH and 4.9% (63/1494) among patients treated with VKA. (S3 Table).

**Fig 4:**
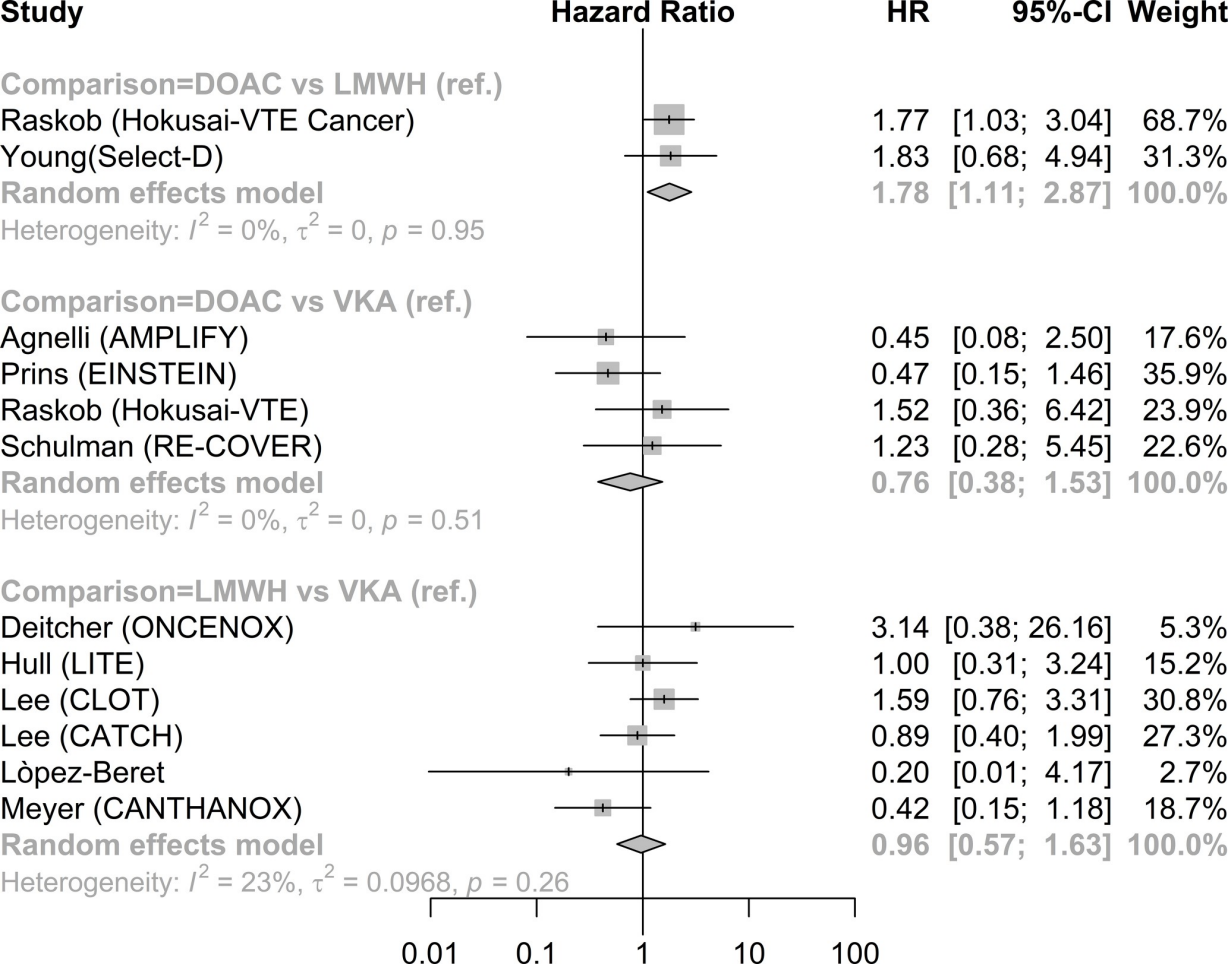
Direct meta-analysis major bleeding forest plot.

Direct comparisons showed a higher rate of major bleeding in patients treated by DOAC compared to LMWH (HR 1.78, 95%CI 1.11 to 2.87, RD 3.0; 95% CI 0.4 to 7.1). (Fig 4 and S4 Table). There was no significant difference in the rate of major bleeding between DOAC and VKA (HR 0.76, 95% CI 0.38 to 1.53) or between LMWH and VKA (HR 0.96, 95%CI 0.57 to 1.63; Fig 4). The network meta-analysis showed no significant difference in major bleeding between treatment groups (Table 2).

### Clinically relevant non-major bleeding

CRNMB was reported in 11 studies including 4400 patients [5, 6, 8, 21, 22, 31, 33–37]. The pooled risk of CRNMB was 12.6% (161/1283) among patients receiving DOAC, 13.8% (203/1698) among those receiving LMWH and 16.5% (217/1419) among those treated with VKA (S3 Table). No significant differences were detected between treatments in direct comparison, as well as in the network meta-analyses (Table 2 and S5 Table).

### Gastro-intestinal bleeding

GI bleeding was reported in 2 studies comparing DOAC to LMWH (1,452 patients) [21, 22] and in 2 studies comparing VKA to LMWH (181 patients) [32, 33]. The pooled risk of GI bleeding was 5.7% (37/725) among patients receiving DOAC, 2.5% (16/815) amongpatients receiving LMWH, and 8.7% (8/93) among those receiving VKA (S3 Table). In direct comparison, the rate of GI bleeding was increased in patients treated with DOAC compared to LMWH (HR 2.88, 95%CI 1.53–5.44, RD 8.7, 95%CI 2.5 to 19.3) and was similar in patients treated with LMWH or VKA (HR 0.42; 95%CI0.12 to 1.48). The network meta-analysis showed no significant difference of GI bleeding when DOAC were compared to VKA (HR 1.22, 95%CI 0.30 to 4.96) or LMWH to VKA (HR 0.42, 95% CI 0.12 to 1.48)(Table 2 and S5 Table).

### Mortality

Twelve studies including 4610 patients reported mortality [5–8, 21, 22, 31, 33–37]. The pooled risk of death was 18.2% (344/1316) among patients receiving DOAC, 33.8% (612/1767) among patients receiving LMWH and 24.9% (439/1527) among patients receiving VKA (S3 Table). There were no significant differences in mortality between treatments in direct comparison, as in the network meta-analysis (Table 2 and S5 Table).

### Study quality and risk of bias

The method of randomisation, allocation concealment and reporting were considered adequate in most studies. Participants were not blinded in most studies [5–8, 21, 22, 31–33, 35]. The risk of bias of individual studies is provided on S6 Table.

### Sources of heterogeneity and sensitivity analyses

A low level of heterogeneity (I2< 25%) was observed in direct comparisons for VTE recurrence, major bleeding and GI bleeding. Moderate heterogeneity (I2 <50%) was observed among studies comparing DOAC to LMWH for mortality and high heterogeneity (I2 > 50%) was observed among studies comparing LMWH to DOAC or VKA for CRNMB (Table 2). Exploration of heterogeneity, publication bias and sensitivity analysis were hindered by the small number of studies in the different comparison arms. The leave-one out meta-analysis did not significantly alter comparison estimates. There were some differences in treatment estimates between direct and indirect evidences (Table 2 and S7 Table) although p-values for comparison were not statistically significant. The proportion of direct evidence in the network analysis varied from 36 to 100%. P-value for inconsistency was statistically significant for major bleeding (S8 Table).

### Treatment ranking

According to the network meta-analysis, DOAC were associated with the highest certainty to be superior to other treatments for VTE recurrence (P-score 0.98) followed by LMWH (P-score 0.51) and VKA (P-score 0.01). LMWH was associated with the highest certainty to be superior to other treatments for safety outcomes, followed by VKA and DOAC (Table 3).

**Table 3:** Ranking of treatments according to network meta-analysis.

	P-score for ranking of treatments		
	DOACs	LMWH	VKA
***Recurrency***	0.986	0.515	0
***Major bleeding***	0.214	0.857	0.429
***CRNMB***	0.261	0.950	0.290
***GI bleeding***	0.196	0.955	0.349
***Mortality***	0.178	0.839	0.482

## Discussion

This meta-analysis suggests that DOAC are an efficacious alternative to LMWH to prevent VTE recurrence in cancer patients. In the present study, DOAC were associated with a lower rate of VTE recurrence compared to LMWH in direct comparison studies.

In the network meta-analysis, treatment effect estimates were also in favour of DOAC and the upper limit of the confidence interval was close to statistical significance. Additionally, the present study confirms the superiority of LMWH over VKA to prevent VTE recurrence.

These results are in accordance with previous meta-analyses and observational studies comparing anticoagulant strategies in cancer patients. [9, 39–41].

While DOAC were shown superior to other anticoagulant strategies to prevent VTE recurrence, we also found an increased rate of major bleeding in patients receiving DOAC compared with those receiving LMWH. Interestingly, estimates from direct and network comparisons were inconsistent as this increased rate of bleeding was observed only in the direct comparison analysis. Similarly, previous meta-analyses reported contrasting conclusions according to their design: direct comparison meta-analyses [9, 40] concluded to an increased rate of major bleeding in patients allocated to DOAC compared to LMWH, while network meta-analyses did not [39, 42].

We believe that network meta-analyses including indirect evidence from studies comparing DOAC to VKA possibly failed to demonstrate this increased rate of major bleeding because these studies included patients with less active or advanced cancer and thus at lower bleeding risk. The rate of major bleeding in the DOAC arm was two to three times lower in studies comparing DOAC to VKA than in studies comparing DOAC to LMWH (S3 Table). Including indirect evidence from studies comparing DOAC to VKA in the network meta-analysis underestimates the overall rate of bleeding for DOAC compared to LMWH. This may artificially favour DOAC in the indirect comparison with LMWH. Therefore, we consider that estimates from direct comparisons studies dedicated to cancer patients should be considered as more appropriate and applicable.

The increased rate of major bleeding in patients allocated to DOAC in studies comparing DOAC to LMWH was mainly due to the higher rate of upper gastro-intestinal bleeding. These events occurred mainly among patients who entered the trials with GI cancer. A safety analysis of the SELECT-D trial after enrolment of 220 patients showed an increased rate of bleeding among patients with oesophageal or gastric cancer and these cancers were subsequently excluded from enrolment. In the Hokusai-cancer study, subgroup analysis showed a significant interaction between GI cancer and increased risk of major bleeding in patients receiving edoxaban.

Mechanisms of DOAC-related gastro-intestinal bleeding could be due to an incomplete intestinal absorption, which can increase the amount of active molecule inside the gastrointestinal lumen and exacerbate existing lesions [43]. Nevertheless, DOAC seem to increase bleeding risk of other mucosa, such as vaginal and upper airways [44, 45]. Direct factor Xa inhibitors also decrease local thrombin generation and platelet activation which may contribute to mucosal bleeding [46]. As the number of studies comparing DOAC to LMWH was limited, we were not able to evaluate if this increased risk of bleeding was class specific or differed between molecules. A population-based study with a propensity-score matched cohort showed that risk of GI bleeding may vary between different Factor Xa inhibitors [47].

Another explanation to the superior efficacy of DOAC to prevent recurrence, and its increased rate of bleeding might be a lower degree of anticoagulation in the LMWH arm. The two studies comparing DOAC to LMWH used a conventional regimen of dalteparin as a comparator. This regimen includes a 25% dose reduction after the first month of anticoagulation, which might contribute to a lower efficacy and improved safety. Finally, the time under treatment was longer among patients allocated to DOAC than those allocated to LMWH, which reflects the better adherence to oral treatment and may also contribute to the lower rate of recurrence and increased rate of bleeding. In real world studies, the differences in adherence to oral or parenteral treatment are even more marked [12, 48, 49] which may further increase safety and efficacy differences.

Overall, DOAC appeared as the most probably superior treatment to prevent recurrence while the LMWH strategy was likely safer for bleeding complications. In terms of absolute differences, the reduction of VTE recurrence with DOAC compared to LMWH and the increase in major bleeding were of similar magnitude precluding to conclude to the superiority of any class of treatment but rather suggesting to tailor the choice to each patient’s thrombotic/bleeding risk profile.

Our study has limitations. First, systematic reviews and meta-analyses rely on the quality of included studies. Although most included studies were considered at low risk of bias, patients were not blinded in most studies. Moreover, the number of included studies comparing DOAC to LMWH and the number of events in some comparison arms were small, which limits the precision of treatment effect estimates and precludes analysing additional sources of heterogeneity.

We compared different classes of anticoagulants but molecules and treatment regimens varied across studies. Unfortunately, the limited number of available studies was insufficient to evaluate a molecule effect within a given class. Nevertheless we pooled the results of these different molecules and regimens without observing a large amount of heterogeneity.

Our study has also several strengths. First, we performed a thorough literature search to provide an exhaustive summary of the current best evidence. Second, in contrast to previous meta-analyses, we reported combined hazard ratios which are measures of association better suited to survival data than risk ratios or odds ratios, especially when lengths of follow-up vary across studies [50]. Third, we aimed to support clinical decision by providing relative and absolute risk differences and treatment ranking for efficacy and safety outcomes. Finally, we provided both estimates for direct and network comparisons in order to take into account direct and indirect evidence and contribute to explain contrasted data observed in previously published systematic reviews on this topic.

## Conclusion

Our meta-analysis shows that DOAC may be more effective than LMWH to prevent VTE recurrence in cancer patients but are associated with an increased risk of major bleeding, especially in patients with GI cancer. Therefore, the choice of the best anticoagulant strategy should be personalised, taking into account recurrence and bleeding risks, including cancer site, and patients’ values and preferences. Results of ongoing studies may help clarify which patients may benefit most from DOAC and potential differences between available Xa inhibitors.

## Supporting information

S1 Table PRISMA checklist. (DOCX)

S2 Table Detailed search strategy. (DOCX)

S3 Table Pooled risks in different treatment arms. (DOCX)

S4 Table Risk differences extrapolated from baseline risk in the Hokusai cancer VTE study. (DOCX)

S5 Table Forrest plots for each outcome. (DOCX)

S6 Table Risk of bias of included studies. (DOCX)

S7 Table Comparison between direct and indirect evidence. (DOCX)

S8 Table Heterogeneity and inconsistency analysis. (DOCX)
