# Supplementary material for: Anticoagulant therapy for acute venous thrombo-embolism in cancer patients: A systematic review and network meta-analysis
Source: PLoS One. 2019 Mar 21;14(3):e0213940. doi: 10.1371/journal.pone.0213940 (PMC6428324; doi:10.1371/journal.pone.0213940)
Supplement: S2 Table — (DOCX) [file pone.0213940.s002.docx]

**S2 Table. Detailed Search strategy**

**Pubmed**

| #1 | Pulmonary embolism [MeSH Terms] OR Venous thrombosis [MeSH Terms] OR venous thromboembolism[MeSH Terms] |
| --- | --- |
| #2 | Apixaban OR dabigatran OR edoxaban OR rivaroxaban OR direct oral anticoagulant OR new oral anticoagulant OR direct antiXa OR direct anti IIa OR direct thrombin inhibitor |
| #3 | Heparin, low molecular weight [MeSH Terms] OR dalteparin OR tinzaparin OR enoxaparin OR nadroparin |
| #4 | Antivitamin K OR warfarin OR acenocoumarol OR phenprocoumon OR coumadin |
| #5 | Randomized controlled trial [MeSH Terms] |
| #6 | #1 AND #2 OR #3 OR #4 AND #5 |

**Central**

| #1 | MeSH descriptor : [Pulmonary embolism] explode all trees |
| --- | --- |
| #2 | MeSH descriptor : [Venous Thromboembolism] explode all trees |
| #3 | MeSH descriptor : [Venous Thrombosis] explode all trees |
| #4 | #1 OR #2 OR #3 |
| #5 | apixaban OR dabigatran OR edoxaban OR rivaroxaban OR betrixaban OR “direct oral anticoagulant” OR “new oral anticoagulant” OR “direct antiXa” OR “direct antiIIa” OR “direct thrombin inhibitor” OR “direct factor Xa inhibitor” in Trials |
| #6 | MeSH descriptor : [Heparin, Low-Molecular-Weight] explode all trees |
| #7 | dalteparin OR tinzaparin OR enoxaparin OR nadroparin in Trials |
| #8 | Antivitamin K OR warfarin OR acenocoumarol OR phenprocoumon OR Coumadin in Trials |
| #9 | #5 OR #6 OR #7 OR #8 |
| #10 | #4 AND #9 |

**Embase**

| #1 | 'venous thromboembolism'/exp OR 'vein thrombosis'/exp |
| --- | --- |
| #2 | 'direct oral anticoagulant agent'/exp OR 'new oral anticoagulant'/exp OR 'dabigatran'/exp OR 'edoxaban'/exp OR 'apixaban'/exp OR 'rivaroxaban'/exp OR 'factorxa inhibitor' |
| #3 | ‘low molecular weight heparin'/exp OR 'dalteparin'/exp OR 'enoxaparin'/exp OR 'tinzaparin'/exp OR nadroparin/exp |
| #4 | 'antivitamin k'/exp OR 'warfarin'/exp OR 'acenocoumarol'/exp OR 'phenprocoumon'/exp OR coumadin/exp |
| #5 | ‘randomized controlled trial’/exp |
| #6 | #2 OR #3 OR #4 |
| #7 | #1 AND #5 AND #6 |
