# Supplementary material for: Anticoagulant therapy for acute venous thrombo-embolism in cancer patients: A systematic review and network meta-analysis
Source: PLoS One. 2019 Mar 21;14(3):e0213940. doi: 10.1371/journal.pone.0213940 (PMC6428324; doi:10.1371/journal.pone.0213940)
Supplement: S4 Table — (DOCX) [file pone.0213940.s004.docx]

**S4. Risk differences extrapolated from baseline risk in the Hokusai cancer VTE study**

|  | **LMWH** | **DOAC** | **DOAC - LMWH** | **VKA** | **VKA - LMWH** |
| --- | --- | --- | --- | --- | --- |
| **Outcome** | **Baseline risk at 1 year** | **Extrapolated risk (95%CI)** | **Risk diff (95%CI)** | **Extrapolated risk (95%CI)** | **Risk diff (95%CI)** |
|  |  |  |  |  |  |
| **Recurrence** | 11.3% | 7.5 (5.3 to 10.4) | -3.8 (-6.0 to -0.9) | 20.2 (15.7 to 25.9) | 8.9 (4.4 to 14.6) |
| **Major bleeding** | 4.0% | 7.0 (4.4 to 11.1) | 3.0 (0.4 to 7.1) | 4.2 (2.5 to 6.9) | 0.2 (-1.5 to 2.9) |
| **CRNMB** | 11.1% | 22.0 (9.0 to 48.1) | 10.9 (-2.1 to 37.0) | 13.4 (8.7 to 20.2) | 2.3 (-2.4 to 9.1) |
| **GI bleeding^1^** | 5.0% | 13.7 (7.5 to 24.3) | 8.7 (2.5 to 19.3) | 11.5 (3.4 to 34.8) | 6.5 (-1.6 to 29.8) |
| **Mortality** | 36.0% | 37.1 (30.0 to 44.8) | 1.1 (-6.0 to 8.8) | 36.3 (31.9 to 40.8) | 0.3 (-4.1 to 4.8) |
|  |  |  |  |  |  |

**^1^Baseline Risk from the LMWH arm SELECT-D study**

**y**
