# Supplementary material for: Anticoagulant therapy for acute venous thrombo-embolism in cancer patients: A systematic review and network meta-analysis
Source: PLoS One. 2019 Mar 21;14(3):e0213940. doi: 10.1371/journal.pone.0213940 (PMC6428324; doi:10.1371/journal.pone.0213940)

**S5 Table. Direct Comparison between treatments, Forest plots for each outcome**

**CRNMB:**


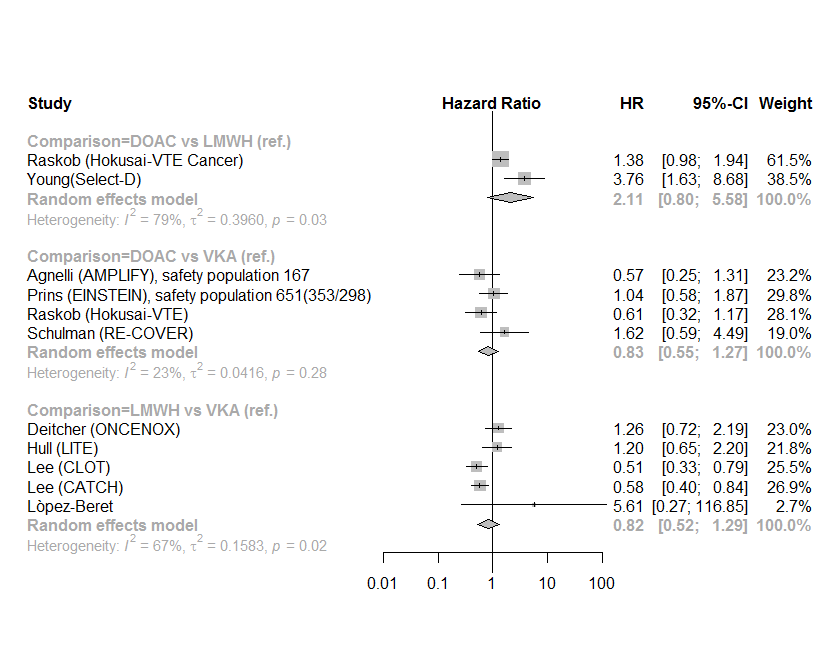


**GI Bleeding:**


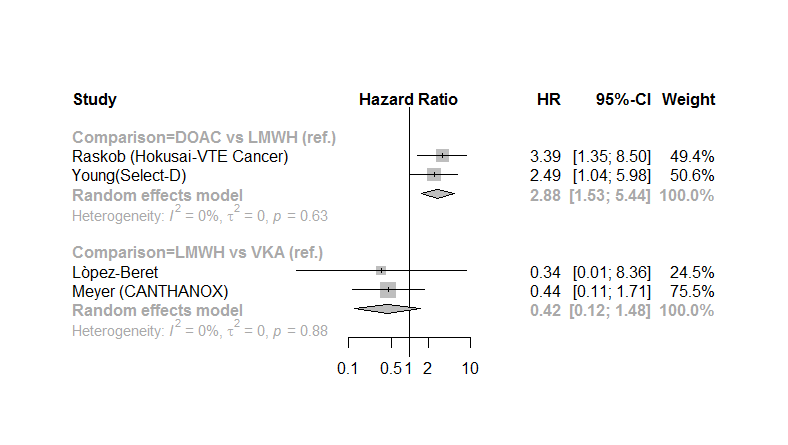
**Mortality:**


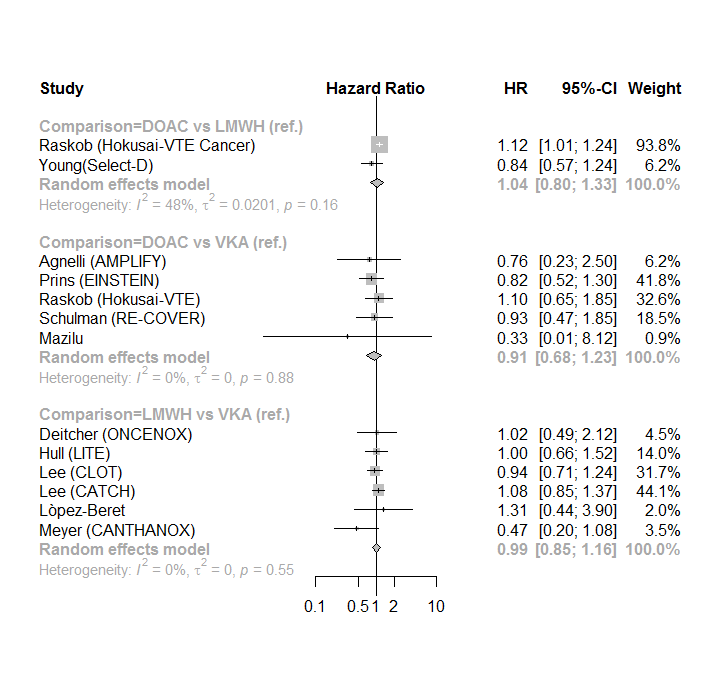

Supplement: S5 Table — (DOCX) [file pone.0213940.s005.docx]
