# Supplementary material for: Anticoagulant therapy for acute venous thrombo-embolism in cancer patients: A systematic review and network meta-analysis
Source: PLoS One. 2019 Mar 21;14(3):e0213940. doi: 10.1371/journal.pone.0213940 (PMC6428324; doi:10.1371/journal.pone.0213940)
Supplement: S6 Table — (DOCX) [file pone.0213940.s006.docx]

**S6 Table. Risk of Bias of included studies**

|  | Randomisation | Allocation concealment | Selective reporting | Other bias | Blinding of participants and personnel | Blinding of outcome assessment | Incomplete outcome data |
| --- | --- | --- | --- | --- | --- | --- | --- |
| Deitcher ONCENOX([33](#_ENREF_33)) | **?** | **?** | **+** | **+** | **-** | **?** | **+** |
| Hull LITE([5](#_ENREF_5)) | **+** | **?** | **+** | **+** | **-** | **+** | **+** |
| Meyer([6](#_ENREF_6)) | **+** | **+** | **+** | **+** | **-** | **+** | **+** |
| Lee CLOT([4](#_ENREF_4)) | **?** | **+** | **+** | **+** | **-** | **+** | **+** |
| Lee CATCH([7](#_ENREF_7)) | **+** | **+** | **+** | **+** | **-** | **+** | **?** |
| Lopez-Beret([35](#_ENREF_35)) | **?** | **?** | **+** | **+** | **-** | **+** | **+** |
| Romera([34](#_ENREF_34)) | **-** | **-** | **+** | **+** | **-** | **?** | **+** |
| Agnelli AMPLIFY([13](#_ENREF_13)) | **+** | **+** | **+** | **+** | **+** | **+** | **?** |
| Mazilu([40](#_ENREF_40)) | **?** | **?** | **?** | **?** | **?** | **?** | **?** |
| Prins EINSTEIN([37](#_ENREF_37)) | **+** | **+** | **+** | **+** | **-** | **+** | **-** |
| Raskob HOKUSAI([38](#_ENREF_38)) | **+** | **+** | **+** | **+** | **+** | **+** | **+** |
| Schulman RE-COVER ([39](#_ENREF_39)) | **+** | **+** | **+** | **+** | **+** | **+** | **?** |
| Raskob HOKUSAI-VTE CANCER([20](#_ENREF_20)) | **+** | **+** | **+** | **+** | **-** | **+** | **+** |
| Young SELECT-D([21](#_ENREF_21)) | **+** | **?** | **+** | **+** | **-** | **+** | **+** |
