# Supplementary material for: Anticoagulant therapy for acute venous thrombo-embolism in cancer patients: A systematic review and network meta-analysis
Source: PLoS One. 2019 Mar 21;14(3):e0213940. doi: 10.1371/journal.pone.0213940 (PMC6428324; doi:10.1371/journal.pone.0213940)
Supplement: S7 Table — (DOCX) [file pone.0213940.s007.docx]

**S7 Table. Comparison between direct and indirect evidence**
