# Supplementary material for: Anticoagulant therapy for acute venous thrombo-embolism in cancer patients: A systematic review and network meta-analysis
Source: PLoS One. 2019 Mar 21;14(3):e0213940. doi: 10.1371/journal.pone.0213940 (PMC6428324; doi:10.1371/journal.pone.0213940)
Supplement: S8 Table — (DOCX) [file pone.0213940.s008.docx]

**S8 Table. Heterogeneity and inconsistency in network meta-analysis**

|  | P-values for | | |
| --- | --- | --- | --- |
|  | Heterogeneity | Inconsistency | Comparison direct vs indirect |
| ***Recurrence*** | 0.967 | 0.124 | 0.124 |
| ***Major bleeding*** | 0.452 | 0.081 | 0.135 |
| ***CRNMB*** | 0.008 | 0.234 | 0.186 |
| ***GI bleeding*** | 0.884 | . | . |
| ***Mortality*** | 0.713 | 0.308 | 0.308 |
